# Internet-based cognitive behavioural therapy for subthreshold depression: a systematic review and meta-analysis

**DOI:** 10.1186/s12888-016-1061-9

**Published:** 2016-10-21

**Authors:** Ting Zhou, Xue Li, Ye Pei, Jianan Gao, Junhui Kong

**Affiliations:** School of Management, Beijing University of Chinese Medicine, #11 North Three-Ring Road East, Chaoyang District, Beijing, China 100029

**Keywords:** Internet-based cognitive behavioural treatment, Subthreshold depression, Randomized controlled trial, Meta-analysis

## Abstract

**Background:**

Subthreshold depression has a considerable impact on individuals’ subjective well-being and psychosocial functioning and is a predictor of major depressive disorder. Internet-based cognitive behavioural treatments (iCBTs) have been used to reduce the symptoms of subthreshold depression. This meta-analysis aims to systematically review evidence indicating the efficacy of iCBT programs on the improvement of depressive symptoms in this population.

**Methods:**

Articles published from January 2005 to July 2016 were searched in the following databases: Medline, PubMed, Web of Science, ScienceDirect, PsycArticles and the Cochrane Central Register of Controlled Trials. Only randomized controlled trials comparing the efficacy of iCBT programs with control groups for participants with subthreshold depression were selected. Both quantitative and qualitative analyses were conducted to examine the efficacy of iCBT interventions.

**Results:**

Tenarticles from 8 randomized controlled trials were identified in this systematic review. The results suggested that iCBT programs had a superior efficacy compared to results from a non-active control group at the post-intervention stage (SMD = − 0.28, CI [− 0.42, − 0.14]; I^2^ = 49 %). However, evidence on the long-term efficacy of iCBT programs is still insufficient and needs further exploration.

**Conclusion:**

There has been substantial evidence that iCBT intervention has a superior short-term efficacy compared to the results of control groups, while its long-term efficacy of iCBT for subthreshold depressive symptoms is inconclusive and must be examined in further research.

**Trial registration:**

The protocol of this review has been registered with the International Prospective Register of Systematic Reviews (PROSPERO), Protocol No. CRD42015023390.

## Background

Depression is a global public health concern. In addition to depression with a full clinical diagnosis, subthreshold forms of depression also exist and are more prevalent [1–3]. Subthreshold depression has been defined in a wide range of forms, varying in the number, severity and duration of depressive symptoms [4, 5]. Generally speaking, people with subthreshold depression score above a certain cut-off in self-rating depression scales or exhibit at least one of the core symptoms for depression as well as one other symptoms, but do not meet the criteria of the Diagnostic and Statistical Manual of Mental Disorders (DSM) for major depression [6, 7].

The results of previous research have shown that the prevalence of subthreshold depression in communities is 7.3 to 23.1 % [8, 9]. The prevalence in some populations, such as the elderly or patients suffering from chronic diseases, might be higher [10].

Although the criteria of major depression are not met, subthreshold depression has a considerable impact on individuals’ subjective well-being and psychosocial functioning [2, 3, 11, 12]. People with subthreshold depression report nearly the same degree of impairment in their health status and functional status as do those with major depression [13, 14]. In addition, subthreshold depression has been considered as a risk factor for the development of major depression and other psychiatric disorders [6, 15]. Therefore, it is necessary to develop appropriate interventions to manage subthreshold depression problems.

Cognitive behavioural therapy (CBT) is one of the main psychological interventions that are used to treat depression. The traditional face-to-face CBT is effective in reducing symptoms of subthreshold depression and preventing major depression and the magnitude of effect size is small to moderate [7, 11, 16–18]. An initial evidence shows that psychological treatments such as CBT tend to be preferred by many individuals with elevated depressive symptoms, compared to medication [19]. However, given the limited access to qualified therapists and relatively high cost, it would be difficult to have face-to-face CBT interventions benefit each individual with subthreshold depression. Moreover, people with mild depressive symptoms might also be less motivated to seek intensive treatment. Based on the stepped care model to manage depression advocated by the National Institute for Health and Clinical Excellence, UK [20], the treatment of individuals with mild to moderate depressive symptoms could start with low-intensity psychological interventions. Internet-based cognitive behavioural therapy (iCBT) is such a less intensive intention compared to the face-to-face therapy.

ICBT programs are designed based on theories of cognitive behavioural therapy and usually include contents of psycho-education, cognitive restructuring, behaviour monitoring, and behaviour activation, among others. These programs are basically self-help interventions which are delivered in text, audio files and video clips. Some programs are entirely self-help programs without any human contact and support, while others involve therapist guidance to generate greater efficacy. Because iCBT programs can be provided anywhere and anytime as long as the Internet is accessible, it is possible to benefit a large number of people who otherwise would not seek treatment. Thus, effective iCBT program is an important addition to traditional face-to-face psychotherapy and an option used in the primary healthcare system.

In terms of the efficacy of iCBT interventions, there is evidence that iCBT is effective in improving symptoms of patients with major depressive disorder [21–25]. It is also superior to CAU (care as usual) alone in reducing mild to moderate depressive symptoms [26]. Interventions with therapist support have greater efficacy against programs without any support [21, 22]. Preliminary evidence shows that guided iCBT interventions could produce equivalent or even greater overall effects for depression as regular face-to-face CBT [27, 28]. Although there have also been a number of randomized controlled trials examining the effectiveness of iCBT programs on reducing depressive symptoms of people with subthreshold depression, the results are inconsistent. Some of them support the effectiveness of iCBT [29, 30], while others show that the improvement of symptoms is not significant [31]. Currently, there are no systematic reviews that evaluate the effectiveness of iCBT programs in improving symptoms in populations with subthreshold depression.

## Objectives

The aim of this systematic review is to evaluate evidence of the effectiveness of iCBT programs on improving the symptoms of subthreshold depression compared to control groups.

## Methods

### Criteria for considering studies for this review

#### Types of studies

Only randomized controlled trials (RCTs) containing at least one group of participants who received iCBT were included in the present study. Single group studies and non-randomized studies were excluded.

#### Types of participants

We included participants with subclinical depressive symptoms, as indicated by elevated depression scores on a standardized depression inventory.

We excluded (1) studies with subjects who were diagnosed with major depressive disorder or dysthymia; (2) studies with subjects reportingsuicidal thoughts; (3) studies involving patients with other primary mental illness, patients with a primary diagnosis of alcohol or drug dependence or patients experiencing psychotic symptoms.

#### Types of interventions

Trials applying various iCBT programs were included in the present study. ICBT programs were defined as interventions based on theories of cognitive behavioural therapy, not conducted at a clinic, and delivered to the participants via the internet.

#### Types of comparators (control groups)

Two types of trials were identified as control groups in the present study: (1) the waiting list control group, in which participants were on the waiting list for treatment and received no immediate intervention; and (2) the low-intensity psycho-educational intervention group, in which participants were provided with some messages regarding depression and stress coping.

#### Types of outcome measures

##### Primary outcomes

Depression levels after the intervention program and during the follow-up were used as the primary outcome variables to indicate the effectiveness of iCBT programs.

##### Secondary outcomes

Because anxiety problems have a high co-morbidity with depression, anxiety symptoms after the interventions and during the follow-up stages were included as secondary outcome variables.

In addition to anxiety levels, general psychological distress, social functioning and quality of life, which were closely correlated with individuals’ mental health, were also used as secondary outcome variables.

##### Data sources and searches

The Cochrane Central Register of Controlled Trials in the Cochrane Library, Medline, PubMed, Web of Science, ScienceDirect and PsycArticles were searched in June 2015. The searches were limited to the years 2005–2015. Additional literature search was done in July 2016 to find the latest articles. The search terms related to subthreshold depression were “subclinical depression”, “subthreshold depression”, “minor depression”, “depressive mood” and “depression”. Intervention related terms were “internet-based cognitive behavioural therapy”, “web-based cognitive behavioural therapy” and “computerized cognitive behavioural therapy”. Another term searched for was “randomized controlled trial”. The initial search was limited to the title, abstract and keywords. Reference lists of recent systematic reviews on iCBT were also manually searched.

##### Data extraction

The results of the various searches were independently reviewed by three authors (GJ, LX, PY). Titles and abstracts were reviewed first to exclude non-RCTs and studies including participants diagnosed with major depressive disorder, dysthymia or other primary mental illnesses. Then, full texts were retrieved, and more details were checked to determine whether the article met the inclusion criteria. Disagreement was resolved by group discussion with another two authors (ZT, KJ). The procedure for the selection of studies was recorded, and reasons for excluding studies were noted.

Four authors (GJ, LX, PY, ZT) participated in the data extraction process. The authors were assigned into small groups to ensure that data from one article was independently extracted by two authors. Disagreement was also resolved by group discussion.

As thefollowing data were extracted:

1. The number and characteristics of participants
2. Descriptions of the intervention group and the control group
3. Symptoms of depression at baseline, the post-intervention stage and the follow-up stage (if applicable)
4. Secondary outcome variables such as anxiety symptoms, psychological distress, social functioning and quality of life at baseline, the post-intervention stage and the follow-up stage (if applicable)
5. Data used to assess the risk of bias, such as the method of randomization, the drop-out number and the method used to handle incomplete data

Depressive symptoms and other outcome variables, including anxiety symptoms and social functioning, were input into RevMan 5.1 for outcome measures.

##### Risk of bias assessment

The quality of randomized controlled trials was assessed using the Cochrane risk of bias tool. As recommended by the Cochrane Handbook for Systematic Reviews of Interventions, sequence generation, allocation concealment, blinding of participants, personnel, outcome assessors, incomplete outcome data, selective outcome reporting, and other threats to validity were assessed independently by two of the four authors (GJ, LX, PY, ZT). Disagreement was resolved by group discussion.

##### Strategies for data analysis

RevMan 5.1 was used to analyse the data. A study-level meta-analysis was performed. The selection of a fixed effect model or a random effect model depended on the results of homogeneity analysis. Continuous data were expressed as a weighted mean difference or a standardized mean difference (SMD), depending on the similarity of scales measuring an outcome. A narrative synthesis was also conducted.

## Results

### Study selection

**Fig. 1:**
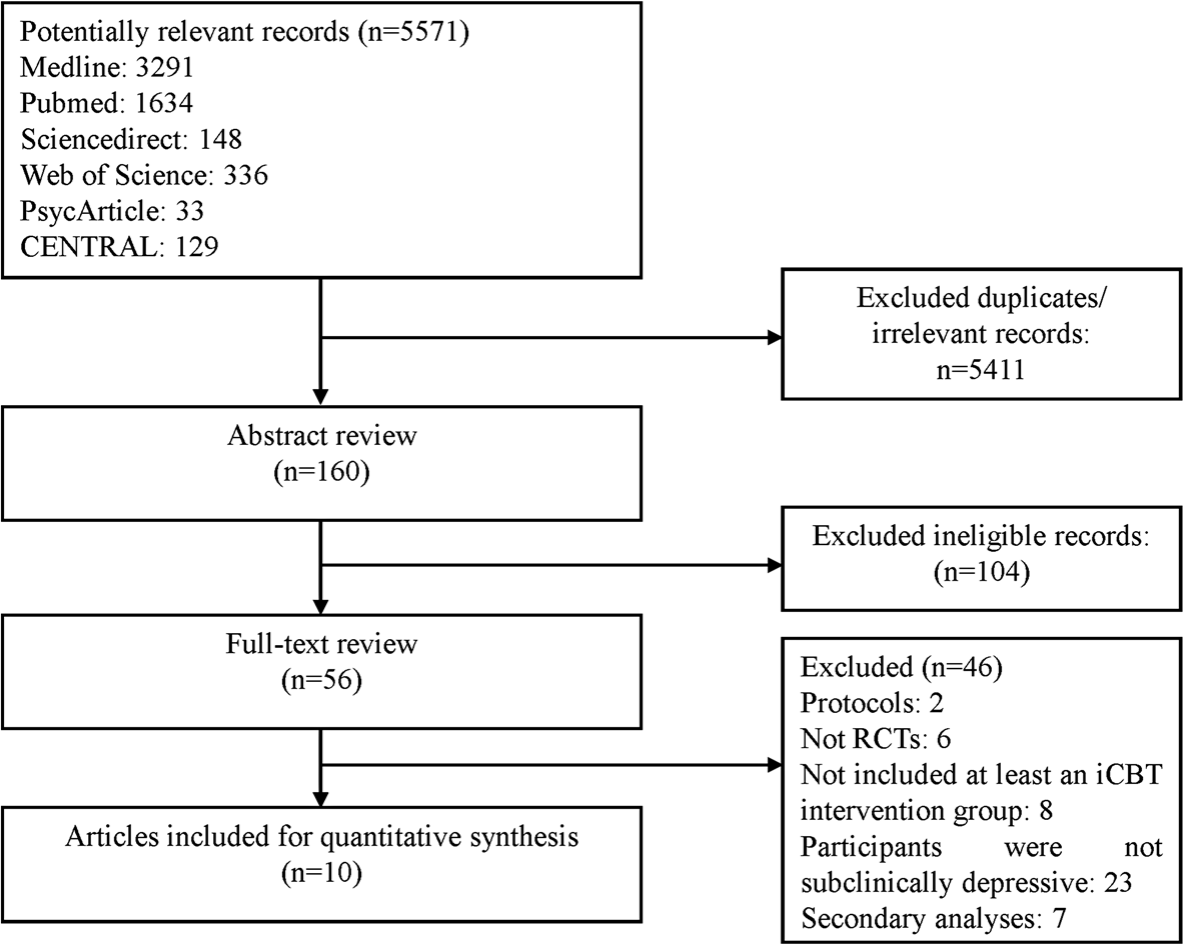
Flow diagram of the study selection process

The initial search produced 5571 potentially relevant records. After removing the duplicates and obviously irrelevant records by reading the titles, 160 articles were identified. An abstract review disqualified 56 of them from the full text review. Finally, 10articles from 8 studies meeting the inclusion criteria were selected for this systematic review. The procedure for study selection and the reasons for exclusion are displayed in Fig. 1.

### Study characteristics

Characteristics of the included studies are listed in Table 1. Three articles from 2 studies recruited elderly participants, one study focused on individuals with chronic pain, and there were no special limits of participants in other studies. The approaches for subthreshold depression screening varied among the studies. In some studies, the subthreshold depression criteria were clearly defined. Only participants who scored above certain depression scale cut-off scores and who did not receive a diagnosis of major depression were included [32–34]. In other studies, although participants with severe depressive symptoms (e.g., suicidal ideation and attempts) and extremely high depression scores were usually excluded, the exclusion of major depression and dysthymia diagnoses was not explicitly reported. The identification of subthreshold depression was determined by checking participants’ depression levels at baseline. We found that depression levels of the participants in these studies were mild to moderate compared with specific depression scale cut-offs. Therefore, we considered that the participants in the included studies were very likely within the range of subthreshold depression.

**Table 1:** Included study characteristics

Source	Participants	Screening of subclinical depression	Conditions	Primary outcome	Secondary outcome	Follow-up (months)	*n* of sessions	Frequency	Support
Buhrman et al. [39]	Patients with chronic pain for more than 3 months	MADRS-S ≥ 10, PRIME-MD	1.iCBT (*n* = 28) 2.WL (*n* = 24)	MADRS BAI PDI	ASI PCS QOLI CPAQ CSQ MPI	12	8	Once a week	Tailored treatment made by therapists; Weekly feedback email on homework from therapists; Weekly reminder from the platform
Buntrock et al. [36]	Employees (>18 years)	CES-D ≥ 16 Without suicidal risk, No MDD and bipolar disorder	1.iCBT (*n* = 202) 2.AC (*n* = 204)	CES-D	SF-12 MCS PCS PSMS PSWQ	6	6	Once or twice a week	Weekly email contact with therapists
Buntrock et al. [40]	Employees (>18 years)	CES-D ≥ 16 Without suicidal risk, No MDD and bipolar disorder	1.iCBT (*n* = 202) 2.AC (*n* = 204)	CES-D	SF-12 MCS PCS PSMS PSWQ	12	6	Once or twice a week	Weekly email contact with therapists
Imamura et al. [34]	Employees (>18 years)	No MDD and bipolar disorder (CIDI)	1.i-CBT (*n* = 381) 2.WL (*n* = 381)	BDI-II	K6 DAS	3,6	6	Once a week	Weekly feedback email on homework from therapists; Weekly reminder from the platform
Mullin et al. [35]	Adults (>18 years)	Self-identified depressive symptoms without suicidal ideas, PHQ-9 ≤ 19, No psychotic mental illness (MINI)	1. iCBT (*n* = 31) 2.WL (*n* = 24)	PHQ-9	GAD-7 K-10 SDS	3	5	Once a week	Weekly email/phone contact with therapists; Weekly reminder from the platform
Phillips et al. [31]	Employees (>18 years)	PHQ-9: Scored 2 or more on five of the 9 items, including Item 1 or Item 2	1.iCBT (*n* = 171) 2.AC (*n* = 188)	WSAS	PHQ-9 CORE10 GAD-7	1.5	5	Once a week	No support
Proudfoot et al. [37]	Adults (18–75 years)	27 ≤ DASS ≤ 63; without suicidal thoughts, attempts and psychotic symptoms	1. iCBT (*n* = 242) 2.AC (*n* = 248) 3.WL (*n* = 230)	DASS-depression	WSAS DASS-anxiety DASS-stress	3	10	Once a week	Weekly phone contact with therapists; Regular reminder from the platform
Spek et al. [25]	Elderly (50–75 years)	EDS ≥ 12 Without psychiatric disorder (CIDI)	1.iCBT (*n* = 102) 2.gCBT (*n* = 99) 3.WL (*n* = 100)	EDS BDI		-	10	Once a week	No support
Spek et al. [33]	Elderly (50–75 years)	EDS ≥ 12 Without psychiatric disorder (CIDI)	1. iCBT (*n* = 102) 2.gCBT (*n* = 99) 3.WL (*n* = 100)	EDS BDI		12	10	Once a week	No support
Titov et al. [41]	Elderly (≥60 years)	Self-identified depressive symptoms	1. iCBT (*n* = 27) 2.WL (*n* = 25)	PHQ-9	GAD-7	3, 12	5	Once a week	Tailored treatment selected by the system

*Note*: *AC* attention control, *ASI* Anxiety Sensitivity Index, *BAI* Beck Anxiety Inventory, *BDI* Beck Depression Inventory, *CES-D* Centre of Epidemiology Studies-Depression, *CIDI* Composite International Diagnostic Interview, *CORE10* Clinical Outcomes in Routine Evaluation, *CPAQ* Chronic Pain Acceptance Questionnaire, *CSQ* Coping Strategies Questionnaire, *DASS* The Depression, Anxiety and Stress Scales, *DAS* The Dysfunctional Attitudes Scale, *EDS* Edinburgh Depression Scale, *gCBT* group cognitive behavioural therapy, *GAD-7* Generalized Anxiety Disorder - 7 items, *GHQ* general health questionnaire, *iCBT* internet-based cognitive behavioural therapy, *K-6* Kessler’s 6-item psychological distress scale, *K-10* Kessler’s 6-item psychological distress scale, *MADRS* Montgomery-Åsberg Depression Rating Scale, *MINI* Mini International Neuropsychiatric Interview, *MPI* Multidimensional Pain Inventory, *PCS* Pain Catastrophizing Scale, *PDI* Pain Disability Index, *PHQ* Patient Health Questionnaire, *PSMS* Pearlln Mastery Scale, *PSWQ* Penn State Worrying Questionnaire,*QOLI* Quality of Life Inventory, *SDS* Sheehan Disability Scales, *SF-12 MCS* SF-12 Health Survey Mental Health Composite Subscale, *SF-12 PCS* SF-12 Health Survey Physical Health Composite Subscale, *WSAS* The Work and Social Adjustment Scale, *WL* waiting list

Several iCBT programs were found in the included studies. The main contents included psycho-education, cognitive restructuring, behavioural activation and other related skills. The included programs lasted 3–10 weeks, with a frequency of one or two sessions per week. Participants usually took the courses at home or other places with Internet access on an individual basis. Some programs are entirely self-help intervention programs, while others are therapist-guided. Online group discussion was included in some studies as well. Examples of these courses include “Coping with depression” [32], “MoodGYM” [31], “e-couch” [30], “The UniWellbeing Course” [35] and “GET.ON Mood Enhancer” [36].

Five studies (6 articles) compared iCBT to the waiting list group only, and 2 studies (3 articles) compared iCBT to the attention control group only, in which participants were provided materials on how to cope with depression. In Proudfoot et al. [37], both the attention control group and the waiting list group were involved [37].

Depression was measured by the BDI (Beck Depression Inventory) in 2 studies (3 articles), the PHQ-9 (Patients Health Questionnaire) in 3 studies (3 articles), the MADRS (Montgomery-Åsberg Depression Rating Scale), CES-D (Centre of Epidemiology Studies-Depression) and the depression subscale of the DASS (Depression, Anxiety and Stress Scales) in 1 study. Secondary outcomes were measured in 7 included studies (8 articles), as listed.

### Risk of bias in the included studies

**Fig. 2:**
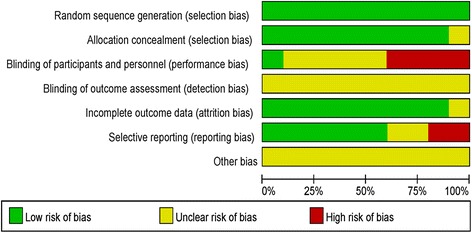
Summaries of risk of bias in included studies

The results of the risk of bias assessment are shown in Fig. 2. Selection bias was assessed by the participant randomization and allocation procedures. The majority of included studies clearly described the randomization methods used and provided adequate information on the randomization concealment. Therefore, the selection bias was generally low.

Performance bias refers to the risk of participants or clinical personnel being aware of the treatment allocation [38]. A high risk of such bias is difficult to avoid in studies involving a self-help psychological intervention group and a waiting list control group. For those studies using a low-intensity intervention control group, such as the attention control group, it is possible to hide the grouping assignment from the participants [31]. In the 8 included studies, participants were directly informed of which groups they were assigned to in 2 studies (3 articles) [36, 39, 40]; grouping assignment was hidden in 1 study [31]; and the rest studies (6 out of 10 articles) did not clearly describe whether the participants and the experimenters were blinded to the condition assignment, leaving the risk of performance bias unclear.

Regarding the attrition bias, it is of great importance because of the relatively high dropout rate of the online intervention programs. Most of the included studies (7 out of 8) reported on efforts made to avoid this bias. For example, characteristics of the participants who did not complete the programs were compared to those of participants who completed the programs in most studies. This procedure ensured that the non-completers did not differ from the completers in terms of demographic features and mental health status at baseline. In addition, missing values were properly imputed in the statistical analyses in most studies. Hence, the risk of attrition bias in most studies was low.

Selective reporting bias was evaluated by comparing the reported outcome variables and those proposed in the protocols. Seven out of 8 studies provided clinical trial registration numbers, and all these research protocols were available. No differences in reported outcomes and proposed outcomes were found in 5 studies.

### Primary outcome: effects on depressive symptoms

#### At the post-intervention stage

**Fig. 3:**
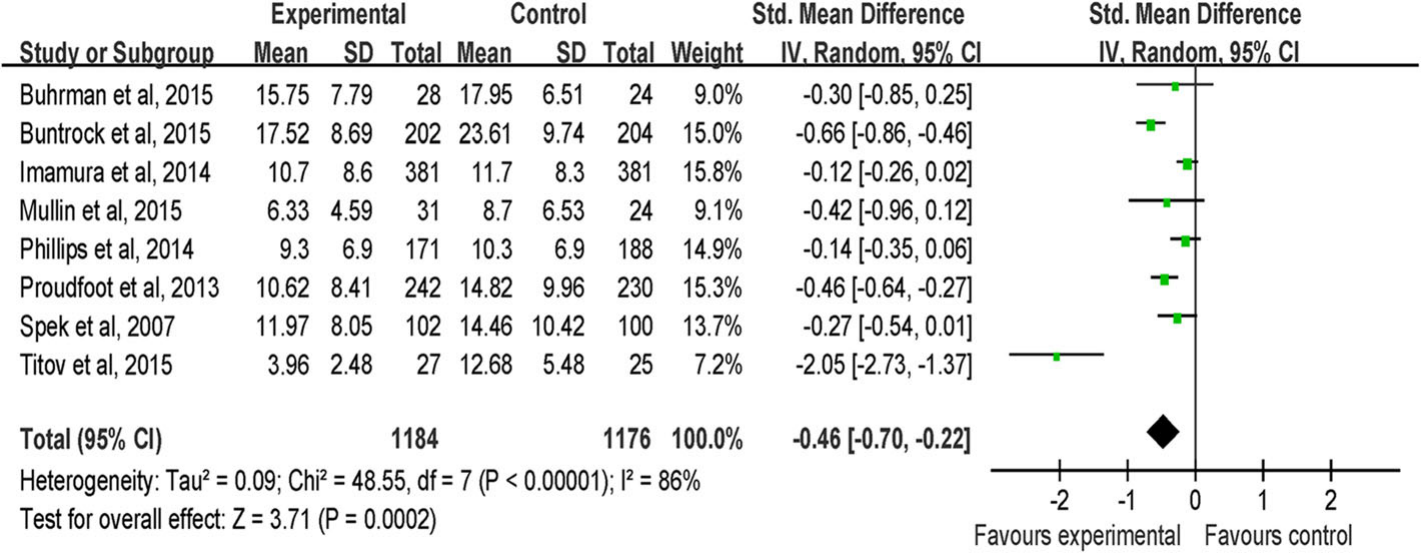
Random effects meta-analysis of iCBT vs. controls for depression at the post-intervention stage

All the eight included studies provided the depression scores of the iCBT groups and control groups at the end of the interventions. The results of homogeneity analysis for these 8 studies revealed considerable heterogeneities (I^2^ = 86 %). As displayed in Fig. 3, a random effects analysis of 8 studies with a total of 2360 participants found a significant treatment effect in favour of iCBT over control groups (SMD = −0.46, CI [−0.70, −0.22]; I^2^ = 86 %).

In 3 studies using the PHQ-9, the effect of iCBT on depressive symptoms was also significant (MD = −2.99, CI [−4.13, −1.86]; I^2^ = 93 %). Further, after excluding one study from the meta-analysis (41) to reduce the heterogeneity, the iCBT group remained superior to the control group in 2 studies using the PHQ-9 (MD = −1.24, CI [−2.54, −0.05]; I^2^ = 0 %).

#### At the follow-up stage

Depressive symptoms of the participants were followed up in all the 8 studies. Both qualitative and quantitative syntheses were conducted to examine the effectiveness of iCBT programs on depression improvement at the follow-up stage.

Depression scores were followed up at 6 weeks after the interventions in the study of Phillips et al. [31]. And researchers reported a non-significant effect of iCBT over the control group at the 6-week follow up.

Depression scores were followed up at 3 months after the interventions in 4 studies [34, 35, 37, 41]. Two of them revealed a significant within-group effect of iCBT interventions on depression improvement at the 3^rd^ month post intervention but did not report the depression scores of the control group at follow-up, leaving the between-group effects unexamined [35, 41]. And the other two studies did not find significant effects of iCBT interventions on reducing depression levels over the control group at the 3-month follow-up [34, 37].

**Fig. 4:**
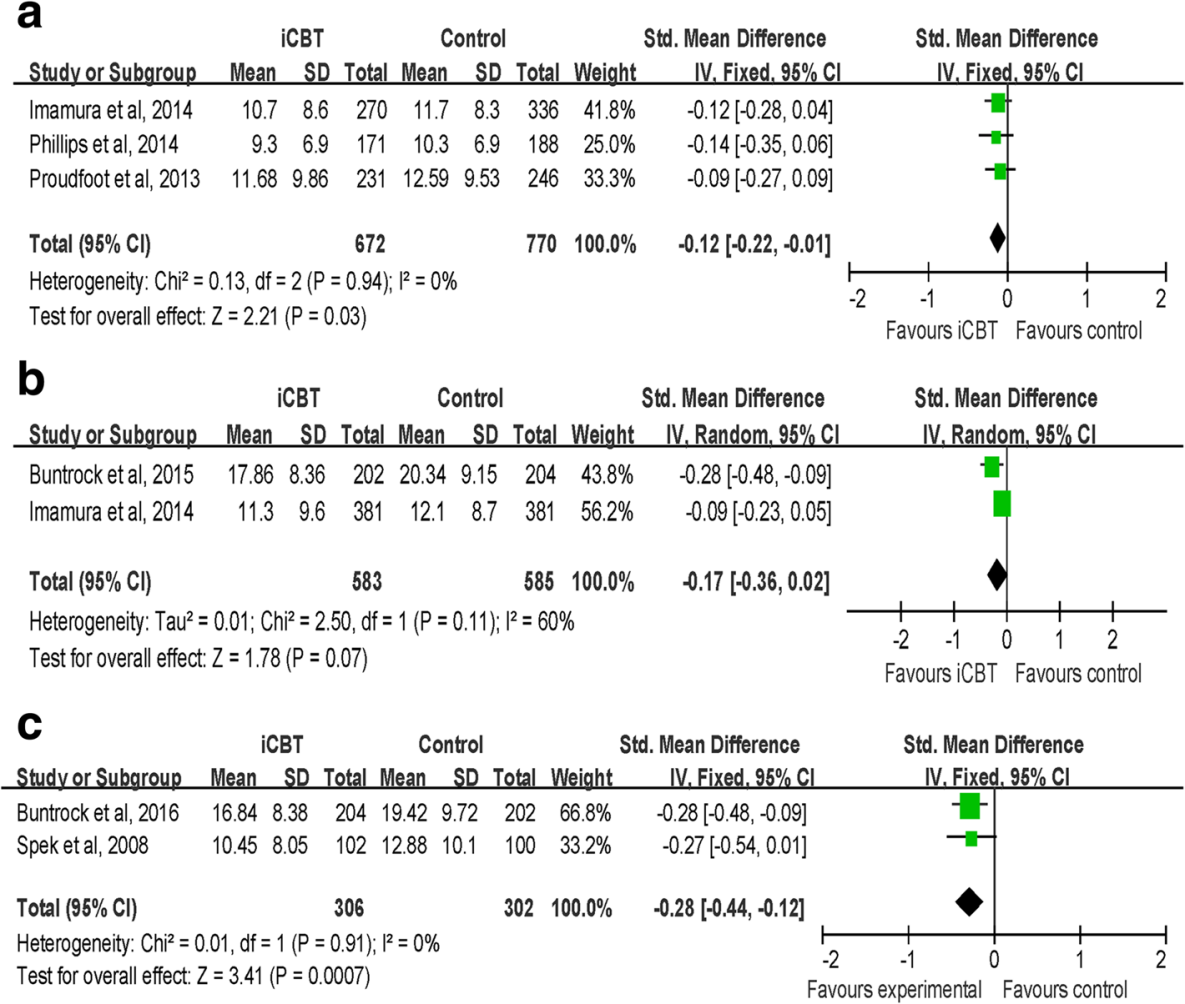
**a** Fix effects meta-analysis of iCBT vs. controls for depression at the at the within 3-month follow-up. **b** Random effects meta-analysis of iCBT vs. controls for depression at the at the 6-month follow-up. **c** Fix effects meta-analysis of iCBT vs. controls for depression at the at the 12-month follow-up

We tried to conduct a meta-analysis by grouping studies following up depression levels of the iCBT group and the control group within 3 months. Three studies were included [31, 34, 37]. As displayed in Fig. 4a, The results indicated that the effect of iCBT intervention on reducing depression levels was significant at the within 3-month follow-up (SMD = −0.12, CI [−0.22, −0.01]; I^2^ = 0 %).

Two studies followed up on the depression scores of participants 6 months post intervention [34]. Results of Buntrock et al. [36] yielded a significant effect of iCBT over the control group while the results of the study of Imamura et al. [34] suggested that the depression levels of iCBT group were not different from that of the control group 6 months post intervention. A random effects analysis of these 2 studies with a total of 1168 participants found the effect of iCBT over the control group was not significant at the 6-month follow-up (SMD = −0.17, CI [−0.36, 0.02]; I^2^ = 60 %) (Fig. 4b).

Depression scores at the 12^th^ month post intervention were available in 4 studies [33, 39, 41]. Two studies only assessed depression scores in the intervention group and the within-group comparison supported the long-term effectiveness of iCBT on reducing depressive symptoms [39, 41]. Studies of Buntrock et al. [40] and Spek et al. [33] provided depression scores of both the intervention group and the control group at follow-up and the between-group comparisons revealed a significant effect of iCBT in both studies. Results of a random effects analysis of these 2 studies indicated that a treatment effect in favor of iCBT over the control group was significant (SMD = −0.28 CI [−0.44, −0.12]; I^2^ = 0 %) (Fig. 4c).

### Secondary outcome: effects on anxiety symptoms, psychological distress, social functioning and the quality of life

#### At the post-intervention stage

**Fig. 5:**
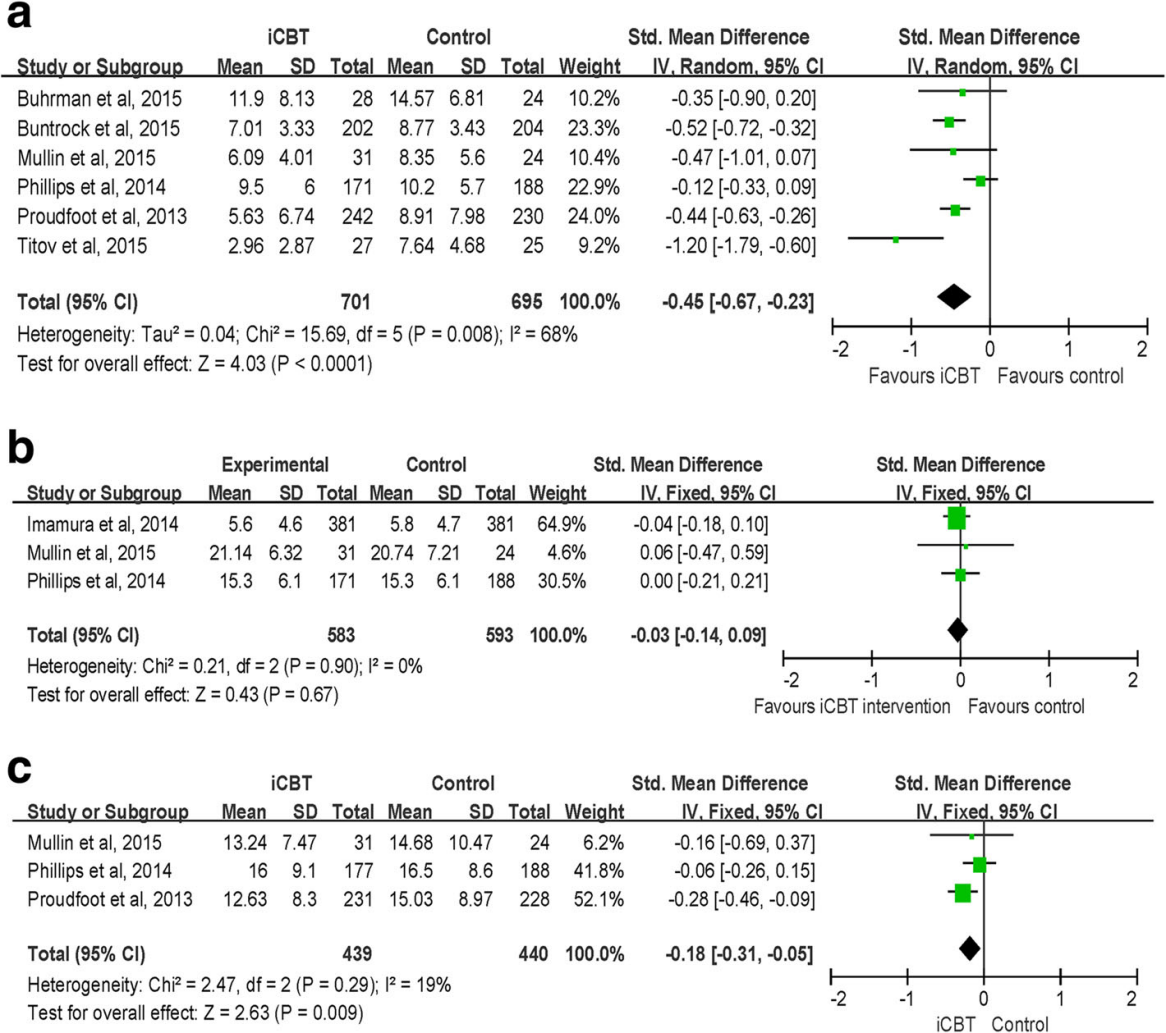
**a** Random effects meta-analysis of iCBT vs. controls for anxiety at the post-intervention stage. **b** Fix effects meta-analysis of iCBT vs. controls for psychological distress at the post-intervention stage. **c** Fix effects meta-analysis of iCBT vs. controls for social functioning at the post-intervention stage

Six studies measured the anxiety symptoms for participants in both the iCBT groups and control groups at the post-intervention stage. A random-effects analysis was conducted with a total of 1396 participants in 6 studies. The results suggested that there was a significant effect favouring iCBT over control groups (SMD = − 0.45, CI [− 0.67, − 0.23]; I^2^ = 68 %) (observed in Fig. 5a). For the 3 studies using the GAD-7 (Generalized Anxiety Disorder-7 items) to measure anxiety, the effect of iCBT on reducing anxiety levels was also significant (MD = − 1.76, CI [− 2.73, − 0.78]; I^2^ = 81 %). After removing one study to reduce the heterogeneity [41], a meta-analysis ofthe remaining 2 studies using the GAD-7 to measure anxiety revealed a non-significant effect of iCBT on anxiety improvement (MD = −0.97, CI [−2.07, 0.13]; I^2^ = 9 %).

General psychological distress was measured as the secondary outcome variablein 3 studies. A fixed-effects analysis was conducted to examine the effect of iCBT on reducing general psychological distress. The result indicated that the treatment effect in favour of iCBT programs over control groups was not significant (SMD = − 0.03, CI [− 0.14, 0.09]; I^2^ = 0 %) (observed in Fig. 5b).

Three studies observedsocial functioning as the secondary outcome. The result of the meta-analysis indicated that iCBT groups had higher scores in social functioning compared with those of the control groups at the post-intervention stage (SMD = − 0.18, CI [− 0.31, − 0.05]; I^2^ = 19 %) (observed in Fig. 5c).

#### At the follow-up stage

Seven studies measured secondary outcomes for 1–2 waves at the follow-up stages, resulting in data for one 6-week follow-up, four 3-month follow-ups, two 6-month follow-ups and two1-year follow-ups. Because of the great variety in measurements as well as the length of time at follow-up, it is inappropriate to run the meta-analysis. Only qualitative synthesis was conducted.

In the short-term follow-up, Phillips et al. [31] measured anxiety symptoms, psychological distress, social functioning and quality of life at the 6^th^ week post- intervention. No significant effects of iCBT on the secondary outcomes were found.

Four studies measured secondary outcomes at the 3^rd^ month after the programs, 3 studies found that the effectiveness of iCBT lasted in the follow-up stage using the within-group comparisons. For example, Proudfoot et al. [37] measured anxiety and social functioning and both of them remained significantly different from the baseline. Similar findings were obtained in Mullin et al. [35], measuring anxiety, psychological distress and social functioning, and in Titov et al. [41], measuring anxiety.

In the studies of Imamura et al. [34] and Buntrock et al. [36], the secondary outcome was measured in the 6^th^ month after the interventions. The results of Imamura et al. [34] suggested that general psychological distress did not differ in the iCBT group and the control group while Buntrock et al. [36] found significant effect of iCBT on reducing anxiety symptoms at the 6-month follow-up.

Secondary outcomes at the 12^th^ month post intervention were available in the study of Titov et al. [41] and Buntrock et al. [40]. In the former study, the researchers measured anxiety scores and found that iCBT had a significant effect on reducing anxiety symptoms at follow-up using within-group comparisons. And the results of the latter study also yielded a significant effect of iCBT on improving anxiety symptoms using between-group comparisons.

## Discussion

This systematic review included 8 studies that examined the efficacy of iCBT programs in individuals with subthreshold depression compared to control groups. As the results indicated, the iCBT programs had a significant effect on the improvement of depressive symptoms in individuals with subthreshold depression at the post-intervention stage compared to control groups. The iCBT programs were also effective in reducing anxiety symptoms and improving social functioning for these individuals. These results were consistent with previous meta-analysis research that focused on patients with major depressive disorder or anxiety disorder [21–25]. That is, the iCBT intervention is not only beneficial for depressive patients with clinical diagnosis but also helpful in improving depressive symptoms in individuals with subthreshold depression.

Although the short-term efficacy of iCBT intervention in improving depressive symptoms was relatively robust, results on the long-term efficacy remained inconsistent. The effectiveness of iCBT programs was followed-up from 6 weeks to one year in the 8 included studies. More than half of the studies supported the efficacy of the iCBT intervention at the 3^rd^, 6^th^ and 12^th^ months after the programs, while two studies did not find advantages in improving depressive and related symptoms at follow-up stages [31]. Notably, 3 studies collected outcome variables at follow-up in the iCBT group but not in the control group and evaluated the effectiveness of iCBT by comparing depressive symptoms at follow-up and at baseline. The potential confounding effect of time could not be eliminated by this within-group comparison. In line with the ideas of Spek et al. [25], we consider results on the long-term efficacy of iCBT programs for subthreshold depressive symptoms to be still inconclusive and in need of further exploration.

Regarding the differences in magnitude of effectiveness of iCBT programs and the inconsistent results in the long-term effects in various studies, the roles of potential moderators are noteworthy. As previous research indicated, therapist support is considered as an important factor that influences the efficacy of iCBT programs. ICBT programs with therapist support have greater efficacy compared to interventions without any professional support [22]. Characteristics of participants are also associated with outcomes of iCBT programs. Female gender and lower dysfunctional attitude could predict better outcomes at post-test and follow-up [30]. Results of effects of pre-treatment depression severity on iCBT effectiveness are controversial. High pre-treatment depression has been reported to be associated with greater efficacy of iCBT interventions in some studies [42, 43], while the opposite results was found in other studies [44]. Despite the overall effectiveness of iCBT intervention on depression has been established, moderator research is relatively few. Identifying predictors and moderators that influence outcomes of iCBT programs would be helpful to design appropriate iCBT interventions for specific target population with clinical or subthreshold depression.

It is also important to pay attention to the dropout rates of the programs. The average dropout rate is 34.5 % in the included studies, which is higher than the dropout rate (21 %) reported in the meta-analysis of Wantland et al. (2004) [45] and the rate (31 %) reported by Melville et al. (2010) [46]. Lack of a therapeutic relationship might be a reason for the high dropout rate. Low exit cost and problems of understanding the programs may also be related to the high dropout rate [22]. Based on the experience of previous research, modifying the course to be appropriate for the target population might be helpful in decreasing the dropout rate [35]. Therapist support is also effective to increase adherence of participants [22].

To summarize, intervention programs developed based on theories and practices of cognitive behavioural therapy are effective in improving depressive and related symptoms for individuals with subthreshold depression. ICBT programs could be easily spread via the Internet and could benefit a great number of people with some depressive symptoms but who have not received a full diagnosis of depression in a convenient way. This type of intervention is a promising method used in primary healthcare and could be effective in preventing the development of major depressive disorder.

There are some limitations in the present systematic review. First, there might be selective bias in the process of study selection. It was not always explicitly stated in the studies whether participants met the criteria of subthreshold depression, and the required information on inclusion and exclusion criteria was sometimes incomplete. Second, subthreshold depression is a spectrum with a wide range. The criteria of subthreshold depression varied across studies, as did the severity of depression levels. These variations might increase the heterogeneity among studies. Third, because of the small sample size, factors influencing efficacy of iCBT programs for individuals with subthreshold depression were not examined in the present study. This is an important research topic that deserves further research attention.

## Conclusion

In conclusion, substantial evidence has been found that iCBT intervention has a superior short-term efficacy compared to control groups for individuals with subthreshold depression. However, the long-term effectiveness of iCBT for subthreshold depressive symptoms is inconclusive and must be examined in further research. ICBT intervention has the potential to be used as an important primary healthcare tool to prevent the development of major depressive disorder.

## Abbreviations

BDI: Beck depression inventory
CAU: Care as usual
CBT: Cognitive behavioural therapy
CES-D: Centre of epidemiology studies-depression
DASS: Depression anxiety and stress scales
DSM: The diagnostic and statistical manual of mental disorders
GAD-7: Generalized anxiety disorder - 7 items
iCBT: Internet-based cognitive behavioural treatment
MADRS: Montgomery-åsberg depression rating scale
MD: Mean difference
PHQ: Patients health questionnaire
RCT: Randomized controlled trail
SMD: Standardized mean difference
